# Molecular characterization of the 2018 outbreak of lumpy skin disease in cattle in Upper Egypt

**DOI:** 10.14202/vetworld.2020.1262-1268

**Published:** 2020-07-04

**Authors:** Ahmad M. Allam, Mohamed Karam Elbayoumy, Eman H. Abdel-Rahman, Ahmed G. Hegazi, Tarek Korany Farag

**Affiliations:** 1 Department of Parasitology and Animal Diseases, National Research Centre, EL Buhouth St., 12622 Giza, Egypt; 2 Department of Zoonotic Disease, National Research Centre, EL Buhouth St., 12622 Giza, Egypt

**Keywords:** fusion gene, lumpy skin disease, P32 gene, phylogeny, Upper Egypt

## Abstract

**Background and Aim:**

Lumpy skin disease (LSD), an infectious disease of cattle, is characterized by raised nodules on the skin. Although the morbidity rate of LSD is low, it has a considerable fatality rate. Despite the annual mass vaccination of livestock with sheep pox vaccine (Veterinary Serum and Vaccine Research Institute, Egypt) enforced by Egyptian authorities, the LSD virus (LSDV) continues to circulate almost every summer. The present study aimed to discover the cause of cows naturally infected with LSDV circulating in Upper Egypt during the summer of 2018 using polymerase chain reaction (PCR) assay and to analyze their phylogenetics against reference genome sequences.

**Materials and Methods:**

We cultured LSDV in specific pathogen-free embryonated chicken eggs (SPF-ECE) and used conventional PCR to identify fusion and P32 genes, previously deposited in GenBank (MN694826, MN694827, and MN954664). Sequencing and phylogenetic analyses were performed on these two highly conserved viral genes.

**Results:**

LSDV infection of SPF-ECE resulted in characteristic white pock lesions. PCR products were identified on 1.5% agarose gel after electrophoresis at the expected positions for the fusion and P32 genes at 472 and 587 bp, respectively.

**Conclusion:**

The present study revealed that the two viral genes were identified from the Beni Suef and Sohag Governorates in all clinical cases and confirmed the circulation of LSDV in this outbreak. After sequencing, these genes were identical to those of the LSDV that had been identified and recorded in GenBank for the past 3 years.

## Introduction

Lumpy skin disease (LSD), caused by the LSD virus (LSDV), infects cattle and is characterized by nodules on the skin. The nodules are round, raised, and painful, and they involve the cutaneous skin and the mucosa of the respiratory tract, eyes, and genital tract. Nodules develop on the muzzle, buccal mucous membranes, nasal cavity, udder, and teats. Edema develops on the legs and brisket, and the regional lymph nodes become enlarged. Secondary infection occurs and causes suppuration, sloughing, and necrosis resulting in hard, raised areas separated from the surrounding skin that forms ulcers, which heal and scar [1].

Historically, LSDV was first documented in Zambia in 1929. Later, it spread into most of Africa and the Middle East [2]. In East Africa, it was first reported in Kenya in 1957 [3], Sudan in 1972, and Somalia in 1983 [4]. It is currently represented in all geographical regions of the African continent, with several outbreaks reported annually [4]. Its occurrence was confirmed in Egypt and Palestinian territories between 1988 and 1989 and again in 2006, 2011, and 2014 in Egypt [5,6]. Incidents of this disease also have been reported in European and West Asian regions [7-9]. In 2015 and 2016, the disease spreads to the Balkans and the Caucasus [10]. In 2014, LSDV began escalating to Turkey; in 2015, it was found near the borders of Greece and was later confirmed in-country [11].

According to the World Animal Health Information Database (https://www.oie.int/en/animal-health-in-the-world/wahis-portal-animal-health-data/), LSD has been traced in Egypt with emergence and disappearance in the summer seasons since 2010. LSDV was first reported in the northeast area (Suez Canal governorates) in the summer of 1988 and spread to most of Egypt’s governorates, leading to high rates of morbidity and mortality among Egyptian cattle [12]. Afterward, the disease reappeared in the summer of 1989, and in a short period, it had spread to most Egyptian governorates [12]. The morbidity rate of the cattle population was low (about 2%), but a considerable mortality rate was recorded [13]. Countries sharing a border with Egypt and those on the Mediterranean coastline remain officially free of LSD [10]. The virus continues to mingle in the Middle East and is considered a potential menace to the Middle East and North Africa area [7,14]. In 1989, suspected cases of LSDV were reported on the Arabian Peninsula in Saudi Arabia (Kingdom of Saudi Arabia [KSA]) in a herd of wild goats [15]. Furthermore, outbreaks were discovered in countries neighboring the peninsula [8,13,16]. It was investigated in many regions of KSA during the fall of 2016 [17].

Despite annual mass vaccination with sheep pox vaccine (Veterinary Serum and Vaccine Research Institute, Egypt) by the Egyptian authorities, LSDV still circulates almost every summer. For instance, during the summer of 2006, an extensive LSD outbreak hits Egypt, affecting 16 provinces [18], and it reemerged in 2011 and 2014 [19,20] leaving small land farmers with painful economic losses and veterinary authorities with inadequate control measures.

House *et al*. [16] reported that only cattle developed the disease, whereas sheep, goats, and water buffalo appeared clinically healthy during the LSD outbreak in Ismailia in 1988. The virus has a limited host range and does not complete its replication cycle in non-ruminant hosts [21]. Different breeds, sexes, and ages are susceptible to the disease, but there is evidence to support young animals that may be more susceptible to the severe form of the disease [8].

LSD is caused by LSDV, which, together with sheep poxvirus and goat poxvirus, belongs to the genus *Capripoxvirus*, subfamily *Chordopoxvirinae*, of the family *Poxviridae* [22]. Diagnosis of LSD depends initially on clinical signs. A definite diagnosis can be made through virus isolation, electron microscopy, or a variety of immunoassays [23]. In addition, gel-based polymerase chain reaction (PCR) is used in detection [24-27].

The current study was performed to detect the cause of the outbreak of LSDV circulating in Upper Egypt during the summer of 2018 from clinically suspected animals using conventional PCR assay as a sensitive diagnostic tool and to investigate their phylogenetic connection with those of published genome sequences.

## Materials and Methods

### Ethical approval

All experimental procedures were performed in accordance with the institutional guidelines of the Animal Research Committee, National Research Centre, Egypt, under protocol number 16/219.

### Study area

Samples were collected from local cattle reared in two governorates located in Upper Egypt, Beni Suef Governorate (29.076°N 31.097°E) located about 120 km south of Cairo, capital of Egypt, on the west bank of the Nile River. The other governorate was Sohag (26.56°N 31.7°E) Governorate located 392 km south of Cairo and 272 km south of Beni Suef Governorate.

### Study period, location and sample collection

From May 2018 to July 2018, skin biopsies ­composed of epidermis, dermis, and subcutis of the nodular skin lesions were collected from local cattle reared in Beni Suef and Sohag Governorates for virus detection by conventional PCR (Table-1). These cattle showed biphasic fever (40°C-41.5°C), depression, inappetence, salivation, and ocular-nasal discharge. Superficial lymph nodes were markedly enlarged, especially the prescapular and precrural lymph nodes. Samples were collected aseptically in 15 mL sterile tubes, transported to the laboratory, and stored at −70°C until use.

**Table-1:** Distribution of samples collected and processed from each governorate.

Number of samples	Beni Suef Governorate	Sohag Governorate
Number of collected samples	35	15
Number of positive by PCR (%)	32 (91.4%)	12 (80%)
Number of positive by inoculation in SPF-ECE (%)	12 (34%)	4 (26.6%)

PCR=Polymerase chain reaction, SPF-ECE=Specific pathogen-free embryonated chicken egg

### Sample preparation

Nodules from each cow were minced using a sterile mortar and were suspended in sterile ­phosphate- buffered saline (PBS) with 10% antibiotic solution. Each tissue homogenate was centrifuged at 1500 ×g for 10 min at 4°C. The clear supernatant of each homogenate was frozen at −70°C until use.

### Virus inoculation into embryonated chicken eggs

The isolation of the field virus from the infected animals was done on 11-day-old specific pathogen-free embryonated chicken eggs (SPF-ECEs) through the chorioallantoic membrane (CAM) route [28]. Briefly, 200 μL from the supernatant fluid of each tissue homogenate was inoculated into five ECEs, incubated for 5 days at 37°C, and then examined daily for characteristic pock lesions on infected CAMs. The infected CAMs were suspended in PBS, minced using sterile pestles, then centrifuged at 1500 ×g for 10 min within a cooling centrifuge. The supernatant fluid of the CAMs was kept at −70°C for further investigation.

### Extraction of viral DNA

DNA of LSDV was extracted from frozen supernatants of the skin lesions (nodules) and pock lesions by GF-1 Tissue DNA Extraction Kit (Vivantis Technologies, Malaysia). The concentration of extracted DNA was done using Nanodrop and the DNA was stored at −20°C.

### Oligos used in the detection of LSDV

PCR primers were chosen from unique LSDV sequences within the genes for viral protein P32 and viral fusion protein [29] (Table-2).

**Table-2:** Oligo’s used in detection of the LSDV by conventional PCR.

Protein coding gene	Oligo’s	Annealing temperature	Amplicon size
Viral protein P32	For: 5’-ATGGCAGATATCCCATTATATGTTA -3’	50^o^C	587 bp
	Rev: 5’-GACGATAATCTAATTACATATG -3’		
Viral Fusion protein	For: 5’-ATGGACAGAGCTTTATCA -3’		472 bp
	Rev: 5’-TCATAGTGTTGTACTTCG -3’		

LSDV=Lumpy skin disease virus, PCR=Polymerase chain reaction

### PCR

PCR reaction was applied (T100™ Thermal Cycler Bio-Rad, Laboratories, Inc., USA) to a total volume of 20 mL using a master mix, specific primers at a concentration of 0.25 mM for each primer of either gene, and the template as recommended by the PCR kit manufacturer (Taq™ iNtRON Biotechnology, South Korea). PCR was run as follows: One cycle of 94°C for 5 min; 34 cycles of 94°C for 1 min, 50°C for 30 s, and 72°C for 5 min followed by one final cycle of 72°C for 5 min.

### Rendering of the PCR products

PCR products were separated by agarose gel electrophoresis using a 100 bp DNA ladder (100 bp DNA Ladder h3 RTU, GeneDirex) as a molecular marker on 1.5% agarose (Agarose superior grade type II, Sisco Research Laboratories Pvt. Ltd., India), containing Red Safe and visualized using ultraviolet light trans-illuminator.

PCR amplicons of proper predicted sizes were gel purified using a DNA gel purification kit (MEGAquick-spin plus).

### Multiple sequence alignment and phylogenetic analysis

Purified PCR products were sequenced by Macrogen Lab Technology (Korea) and assembled using ChromasPro software (ChromasPro 1.7, Technelysium Pty Ltd., Tewantin, Australia). Sequence alignment and construction of the phylogenetic tree were conducted to detect the genetic relatedness of the tested strains compared to other worldwide strains registered in the GenBank.

The amplicon produced from the Beni Suef Governorate was directly sequenced in both directions for both genes. The amplicon produced from the Sohag Governorate was directly sequenced in both directions of the fusion gene and one direction of the P32 gene. Sequencing was done with the same primers used to generate the PCR amplicons. The nucleotide sequence data obtained after genetic analysis in DNA Analyzer was compared, aligned, and analyzed using BioEdit Software Program V.5.0.9, USA [30].

Phylogenetic analysis was performed by constructing a neighbor-joining (NJ) tree of the nucleotide sequence data using the MEGA 7 software program [31]. The consistency of the phylogenetic associations was estimated using non-parametric bootstrap analysis with 1000 replicates for NJ analysis. The obtained sequences were submitted in GenBank and then compared with those available in the GenBank database by NCBI BLAST (http://blast.ncbi.nlm.nih.gov/Blast.cgi).

## Results

### Clinical signs

**Figure-1:**
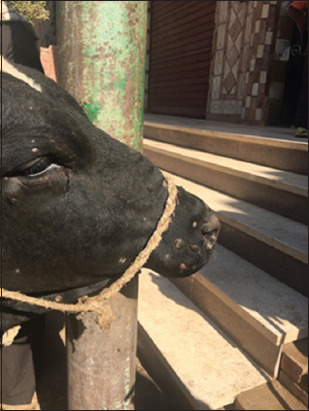
Cattle head showing lumpy skin disease infection scars in the late stage.

**Figure-2:**
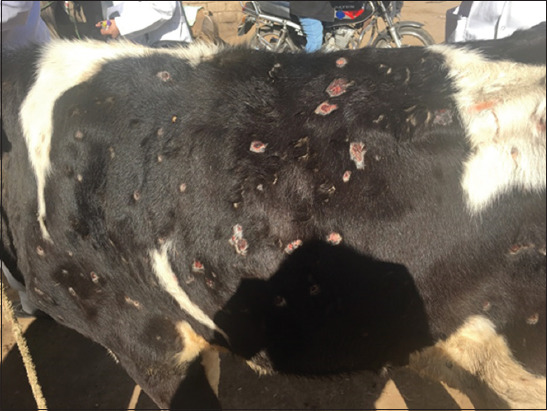
Calf skin showing lumpy skin disease infection scars in the late stage.

**Figure-3:**
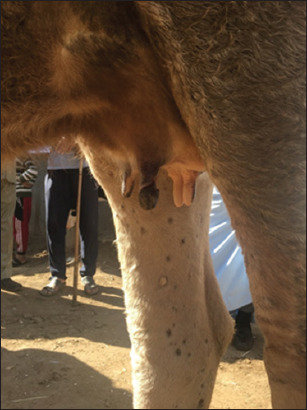
Cow teat showing complete dry of one teat due to lumpy skin disease infection.

LSD had characteristic features beginning with a high fever (40°C-41.5°C) and characteristic nodules raised over the skin with specific globular shape 1-5 cm in diameter. The nodules coalesced together and formed large batches with an irregular border shape. The nodules were also present on the mucous membranes of the nasal cavity, gastrointestinal tract, eyes, udder, teats, legs, and genitalia. The nodules were fibrotic in nature and sometimes ruptured with orange suppuration, leaving a necrotic wound. The skin detached, leaving ulcers and scar formation. LSD also causes edema of abdomen, udder, and genitalia. Edema on male genitalia is dangerous, causing fatality due to the prevention of urination. The animals become lethargic, inappetent, and recumbent (Figures-1-3).

### Isolation of LSDV on SPF-ECE through CAM

**Figure-4:**
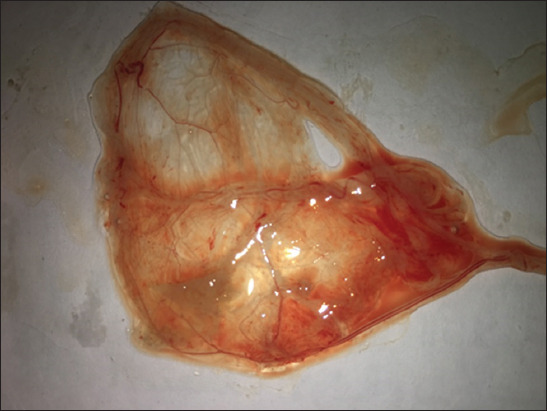
Pock lesion of lumpy skin disease virus on chorioallantoic membrane shows small white foci.

Virus propagation was performed in 11-day-old SPF-ECEs through the CAM. After 5 days of incubation and daily examination for non-specific death, characteristic pock lesions on infected CAMs were observed (Figure-4).

### Detection of LSDV DNA by PCR

**Figure-5:**
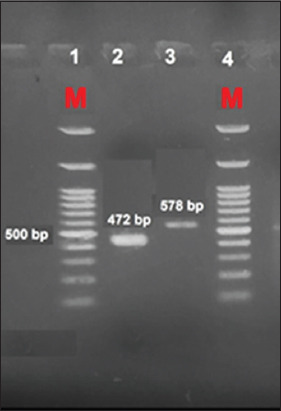
Gel electrophoresis separation of fusion and P32 genes detected in infected samples. DNA ladder (100 bp DNA Ladder h3 RTU, GeneDirex).

DNA bands of LSDV isolates were visualized using an ultraviolet transilluminator after the agarose gel was stained with Red Safe. The amplification products were identified on 1.5% agarose gel electrophoresis at the expected positions for the fusion gene at 472 bp and for P32 at 587 bp (Figure-5).

### GenBank accession

The PCR products of both genes and isolates were deposited in GenBank with accession numbers. The accession numbers for nucleotide sequences of isolates from Beni Suef Governorate are MN694826 for the fusion-coding gene and MN694827 for the P32 coding gene. The accession number for the isolates from the Sohag Governorate is MN954664 for the P32 coding gene.

### LSDV sequencing and phylogenetic trees

The nucleotide sequences of the fusion and P32 genes isolated from Beni Suef and Sohag Governorates during the summer of 2018 were similar with more than 99% homology to that of previously registered LSDV isolates from Egypt and neighboring countries in Africa and Asia.

**Figure-6:**
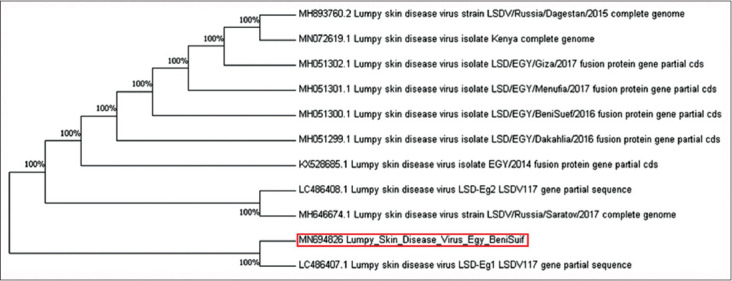
Neighbor-joining tree illustrating phylogeny of lumpy skin disease virus isolated from Beni Suef Governorate based on fusion gene.

**Figure-7:**
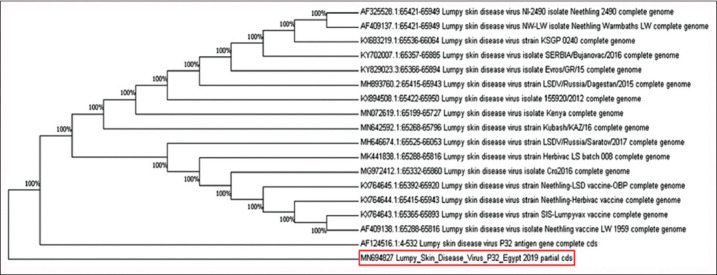
Neighbor-joining tree illustrating phylogeny of lumpy skin disease virus isolated from Beni Suef Governorate based on P32 gene.

The Beni Suef LSDV isolates of the fusion gene recorded similarities of 100% with the previous accession numbers MH051299, MH051300, MH051301, and MH051302 (Figure-6). Beni Suef LSDV isolates of the P32 gene demonstrated similarities of 100% with deposited isolates from Russia (MH646674) and Kenya (MN072619) (Figure-7).

**Figure-8:**
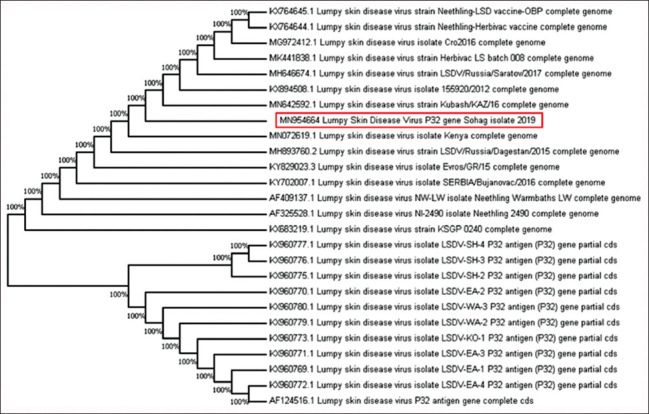
Neighbor-joining tree illustrating phylogeny of lumpy skin disease virus isolated from Sohag Governorate based on P32 gene.

Oligonucleotide primers of the fusion gene succeeded in detecting the genomic DNA of the Sohag isolates, but the sequencing failed with the same primers of the PCR. However, Sohag isolates of the P32 gene recorded similarities of 100% and in the same cluster with deposited sequences from Australia (AF124516) and Iran (KX960770, KX960772, KX960775, KX960776, KX960777, and KX960778) (Figure-8).

## Discussion

In the 2018 outbreak of LSD in Egypt, the disease appeared with a vigorous clinical picture, including a high mortality rate and substantial economic losses of meat and milk. Male cattle showed sterility, and the females presented infertility consistent with previous records [13,32]. In the present study, isolation of LSDV revealed characteristic pock lesions on the CAM of SPF-ECEs, which appeared as pock lesions of distributed foci characteristic of LSD, in agreement with El-Tholoth and El-Kenawy [20].

In the current study, genes detected in samples collected from the Beni Suef and Sohag Governorates were identified and confirmed by conventional PCR. The results agree with the previous studies [24,26,29], emphasizing that PCR is a fast and accurate method to detect LSDV during emergencies. Sequences revealed that all isolates were related to LSDV with similarities of 100%. The LSDV fusion gene sequence of the Beni Suef isolates was fully identical to that in Egypt and was recorded in GenBank with accession numbers MH051299, MH051300, MH051301, and MH051302 as reported from Dakahlia, Beni Suef, Menufya, and Giza Governorates, respectively, through the past 5 years [1,33,34]. The LSDV nucleotide sequence of Beni Suef Governorate isolates was identical to those of the LSDV P32 gene isolated from Russia (MH646674) [11] and Kenya (MN072619) [35].

The Sohag isolates confirmed with the P32 coding gene recorded similarities of 100% and were in the same cluster with deposited sequences from Australia (AF124516) [36] and Iran (KX960770, KX960772, KX960775, KX960776, KX960777, and KX960778) [9]. All of these isolates were in complete similarity with other Egyptian isolates in the past 3 years.

## Conclusion

Isolation of the virus from the CAMs of chicken embryos was an asset in diagnosis but was insufficient for a complete diagnosis. PCR assay is sensitive to identify LSDV. Sanitary controlled measures to LSDV outbreaks are required to improve the understanding of disease epizootiology in Egypt. Furthermore, periodic viral sequence comparisons and phylogenetic analyses of the current LSDVs are necessary for identifying new circulating strains, observing the spread of viruses, and choosing suitable vaccines as a prophylactic measure against viral infection.

## Authors’ Contributions

All authors contributed to the main conceptual design and drafted the manuscript. MKE prepared the samples and data analysis. AMA did the purification of PCR products and run the PCR. AGH and EHA set up manuscript proposal and reviewed results. TKF collected samples and animals follow-up. All authors read and approved the final manuscript.
